# Predictors of improvement in left ventricular systolic function after catheter ablation in patients with persistent atrial fibrillation complicated with heart failure

**DOI:** 10.1186/s12872-024-03840-z

**Published:** 2024-03-23

**Authors:** Xinliang Zhao, Xiaoqin Hu, Wei Bao, Shuo Huang, Fei Li, Chen Liu, Liqi Ge, Quan Zhang, Chaoqun Zhang, Chengzong Li

**Affiliations:** Department of Cardiology, The Affiliated Hospital of Xuzhou Medical University, Xuzhou, 221000 Jiangsu China

**Keywords:** Atrial fibrillation, Heart failure, Tachycardiomyopathy, Radiofrequency catheter ablation, Left ventricular end-diastolic diameter, Low voltage zones

## Abstract

**Aims:**

The current management of patients with atrial fibrillation (AF) and concomitant heart failure (HF) remains a significant challenge. Catheter ablation (CA) has been shown to improve left ventricular ejection fraction (LVEF) in these patients, but which patients can benefit from CA is still poorly understood. The aim of our study was to determine the predictors of improved ejection fraction in patients with persistent atrial fibrillation (PeAF) complicated with HF undergoing CA.

**Methods and results:**

A total of 435 patients with persistent AF underwent an initial CA between January 2019 and March 2023 in our hospital. We investigated consecutive patients with left ventricular systolic dysfunction (LVEF < 50%) measured by transthoracic echocardiography (TTE) within one month before CA. According to the LVEF changes at 6 months, these patients were divided into an improved group (fulfilling the ‘2021 Universal Definition of HF’ criteria for LVEF recovery) and a nonimproved group. Eighty patients were analyzed, and the improvement group consisted of 60 patients (75.0%). In the univariate analysis, left ventricular end-diastolic diameter (*P* = 0.005) and low voltage zones in the left atrium (*P* = 0.043) were associated with improvement of LVEF. A receiver operating characteristic analysis determined that the suitable cutoff value for left ventricular end-diastolic diameter (LVDd) was 59 mm (sensitivity: 85.0%, specificity: 55.0%, area under curve: 0.709). A multivariate analysis showed that LVDd (OR = 0.85; 95% CI: 0.76–0.95, *P* = 0.005) and low voltage zones (LVZs) (OR = 0.26; 95% CI: 0.07–0.96, *P* = 0.043) were significantly independently associated with the improvement of LVEF. Additionally, parameters were significantly improved regarding the left atrial diameter, LVDd and ventricular rate after radiofrequency catheter ablation (all *p* < 0.05).

**Conclusions:**

The improvement of left ventricular ejection fraction (LVEF) occurred in 75.0% of patients. Our study provides additional evidence that LVDd < 59 mm and no low voltage zones in the left atrium can be used to jointly predict the improvement of LVEF after atrial fibrillation ablation.

**Supplementary Information:**

The online version contains supplementary material available at 10.1186/s12872-024-03840-z.

## Introduction

Tachycardiomyopathy (TCM) with left ventricular systolic dysfunction is a rare but treatable cause of heart failure, and atrial fibrillation is the most common cause of TCM in adults [1]. These two diseases often coexist and share common pathophysiological mechanisms and underlying risk factors [2, 3]. According to some research [4, 5], the proportion of AF in patients with HF is between 10% and 57%, while the rate of HF in patients with AF is above 50%. In contrast to other diseases that can cause heart failure, AF-TCM is a reversible disease with a good prognosis after standardized treatment. Therefore, the diagnosis of AF-TCM has extremely important clinical significance. However, at present, TCM is often confused with dilated cardiomyopathy (DCM) because there are not unified diagnostic criteria and the diagnosis is usually retrospective. Predicting which patients will restore LV systolic function after successful AF treatment remains a clinically significant question. Given the poor prognosis of AF with HF, developing effective and safe treatment strategies is crucial. Since most conventional antiarrhythmic drugs (AADs) are contraindicated, often ineffective, or poorly tolerated in patients with heart failure with reduced ejection fraction (HFrEF), catheter ablation of AF provides an increasingly important option for these patients [6, 7]. Several observational and randomized studies [8–11] have provided substantial evidence of high success rates in sinus rhythm maintenance after AF ablation, as well as significant clinical improvement in LV function. In this study, we conducted a retrospective study to evaluate predictors of improved LV systolic function after ablation in patients with AF complicated with HF.

## Methods

### Study population

This was a single-center retrospective clinical observation study. A total of 435 persistent AF patients who underwent catheter ablation of atrial fibrillation in the Affiliated Hospital of Xuzhou Medical University from January 2019 to March 2023 were systematically enrolled; among them, 106 (24.4%) were complicated with heart failure. According to the 2021 ESC guidelines, Heart failure in this study refers to HFrEF (reduced LVEF is defined as ≤ 40%) and HFmrEF (LVEF between 41% and 49%) [12]. After excluding congenital heart disease, valvular heart disease and patients with incomplete data, a total of 80 patients were included in this study. The study protocol was reviewed by the Ethics Committee of the Affiliated Hospital of Xuzhou Medical University, and all patients signed informed consent before surgery.

### Ablation strategy

Consistent with the current guidelines, circumferential pulmonary vein isolation (CPVI) was the first step in all patients and was achieved by circumferential ablation around the PV ostia (recommended power 30–40 W; CF 5–30 g). If AF did not terminate to SR, electrical cardioversion was used to convert into SR after CVPI. During sinus rhythm, a detailed 3-D voltage map of the left atrium was created by using a force-sensing ablation catheter (local contact force > 5 g and > 150 points). All procedures were guided by CARTO (Biosense Webster) electroanatomic mapping system and ablation was performed using open irrigated catheters with contact force (CF) sensing (Thermocool SmartTouch, Biosense Webster). Low voltage areas were defined as areas with amplitudes less than 0.4 mV in more than 3 adjacent low voltage points with a space difference of 0.5 cm [13–16]. If the patient has low voltage areas in the left atrium, we will further measure the area of low voltage and the size of the left atrial area. The proportion of the area of the low voltage areas to the total area of the left atrium was calculated. Additional ablation strategies, including linear lesions, were performed in patients with LVZs after CPVI.

### Epicardial adipose tissue volume

All patients completed Computed Tomography Angiography (CTA) of the left atrial pulmonary vein before CA to exclude the existence of left atrial thrombus, and the Epicardial Adipose Tissue (EAT) volume was measured. First, all data were sent to (AW GE 3.2 United States workstation) for postprocessing of the images. Axial images from the pulmonary artery bifurcation to the root tip were reconstructed semiautomatically from adjacent 0.625 mm sections. After that, the cardiac wall and pericardial dirty layer were found by combining the slice method with the threshold method, the epicardial boundary was delineated layer by layer, the CT value of -50 HU∼-200 HU was set as adipose tissue, and the total EAT volume was automatically calculated [13].

### Follow-up

Antiarrhythmic medications were recommended during a blanking period of 3 months after CA. Generally, we routinely perform 12-ECG or 24-h Holter monitoring to evaluate the recurrence of AF at 1, 3, 6 and 12 months after successful ablation. The recurrence of AF was defined as atrial tachyarrhythmia lasting more than 30 s after CA for three months. The structural and functional changes in cardiac tissue were evaluated by TTE at the 1,3 and 6 months after ablation. Changes in LVEF at any of the three follow-up visits that met our criteria for improvement were placed in the improvement group.

### Statistical analysis

Normally distributed measured data are expressed as the mean ± sd: if they did not fit into a normal distribution, they were described by the median (interquartile spacing) or M (Q25, Q75), and the count data were described by the number (percentage). Count data were also compared by t test or rank-sum test (not normally distributed), and categorical variables were compared by chi-square test or Fisher’s exact test. Logistic regression with postoperative ejection fraction improvement as a dichotomous variable was used to identify factors associated with outcome (independent variables), first in a univariate fashion, then using a backward stepwise selection process. All variables related to LVDd, left atrial low voltages, age, e-GFR and H-LDL with a *P* value < 0.10 were included in the multivariate model. *P* values < 0.05 were considered statistically significant. The statistical analysis of this study was performed using SPSS 26.0 software and R language 4.3.0 software.

## Results

In this research, 80 patients with AF combined with heart failure (LVEF < 50%) were included. According to the postoperative TTE at 6 months, the patients were divided into two groups, and the ejection fraction improvement group (I group) was defined as (i) in the case of baseline LVEF between 40% and 49%: LVEF improvement to more than ≥ 50% and (ii) in the case of baseline LVEF < 40%: ≥ 10% absolute increase from baseline LVEF and LVEF > 40% [17]. The nonimproved group (NI group) did not meet the above criteria (NI group). There were 60 (75.0%) patients in the I group and 20 (25.0%) patients in the NI group. The baseline characteristics of the patients in both groups are described in Table 1. We found that the NI group patients were older than the I group patients, and there were no differences in AF duration, sex, comorbidities or medications between the two groups.

**Table 1:** Baseline characteristics of the patients

	I group(*n* = 60)	NI group(*n* = 20)	P -value
Clinical data			
Age (years)	59(50,66)	67(57,75)	**0.013**
Male n(%)	42(70.0)	12(60.0)	0.408
Height (cm)	168.9 ± 8.6	165.1 ± 9.9	0.103
Weight (kg)	74.7 ± 13.0	70.0 ± 14.3	0.136
BMI (kg/m^2^)	26.07 ± 3.29	25.28 ± 3.26	0.353
AF duration (months)	3.0(1.0,24.0)	4.0(1.0,24.0)	0.505
Past history			
Hypertension n(%)	24(40.0)	8(40.0)	1
Diabetes n(%)	10(16.7)	2(10.0)	0.718
CHD n(%)	16(26.7)	5(25.0)	0.883
Previous stroke n(%)	11(18.3)	5(25.0)	0.747
Smoker n(%)	12(20.0)	3(15.0)	0.869
Drinker n(%)	13(21.7)	4(20.0)	1
Medication			
ACEI/ARB/ARNI n(%)	46(76.7)	15(75.0)	0.879
$\beta$-block n(%)	53(88.3)	16(80.0)	0.574
Diuretic n(%)	44(73.3)	13(65.0)	0.476
SGLT-2i n(%)	10(16.7)	2(10.0)	0.718
Digoxin n(%)	12(20.0)	3(15.0)	0.869

I group = improve group, NI group = nonimprove group, CHD = coronary heart disease, ACEI = angiotensin-converting enzyme inhibitor, ARB = angiotensin receptor blocker, ARNI = angiotensin receptor neprilysin inhibition, SGLT-2i = sodium-dependent glucose transporters 2 inhibitor,

Compared to NI group patients, I group Patients had a significantly smaller LVDd (54.30 ± 5.02 mm vs. 58.95 ± 6.66 mm, *P* = 0.001) and had fewer low voltage areas of the left atrium (19, 31.7% vs. 14, 70.0%, *P* = 0.003). There was no significant difference in the epicardial fat volume, laboratory parameters, or postoperative recurrence of atrial fibrillation (Table 2).

**Table 2:** Epicardial fat volume, echocardiographic, and laboratory parameters of study patients

	I group(*n* = 60)	NI group(*n* = 20)	P -value
TTE			
LVEF(%)	41.4 ± 4.8	43.3 ± 5.0	0.131
LAD(mm)	45.4 ± 4.5	45.7 ± 4.7	0.822
E/A (m/s)	1.17 ± 0.42	1.19 ± 0.44	0.084
LVDd(mm)	54.30 ± 5.02	58.95 ± 6.66	**0.001**
Laboratory parameters			
NT-pro BNP(pg/ml)	2228(1251,3850)	2350(608,5994)	0.731
Hs-CRP(mg/dL)	1.25(0.50,2.98)	1.10(0.50,5.78)	0.946
e-GFR(ml/min/1.73${m}^{2}$)	101.4(82.5,119.9)	91.2(69.0,100.7)	**0.008**
Creatinine(u mol/L)	69.25 ± 14.90	76.90 ± 15.13	0.051
Hb(g/L)	145.7 ± 17.2	138.2 ± 18.5	0.099
TC(mmol/L)	3.97 ± 0.87	3.73 ± 0.82	0.286
TG(mmol/L)	1.17(0.93,1.54)	1.13(0.90,1.78)	0.881
LDL-C(mmol/L)	2.39(1.73,2.74)	2.18(1.49,2.75)	0.519
HDL-C(mmol/L)	1.02 ± 0.22	0.93 ± 0.17	0.089
Epicardial adipose tissue			
Total-EAT volume (cm^3^)	106.22(88.44,133.08)	101.02(85.81,145.41)	0.885
LA-EAT volume (cm^3^)	26.08(18.51,30.91)	28.54(19.92,40.47)	0.386
LVZs n(%)	19(31.7)	14(70.0)	**0.003**
Recurrence of AF n(%)	11(18.3)	4(20.0)	0.869

I group = improve group, NI group = nonimprove group, LA-EAT = left atrial epicardial adipose tissue, TTE = transthoracic echocardiogram, LVEF = left ventricular ejection fractions, LAD = left atrial diameter, LVDd = left ventricular end diastolic diameter, LVZs = low voltage zones

### Predictor of improvement in left ventricular ejection fraction

**Fig. 1:**
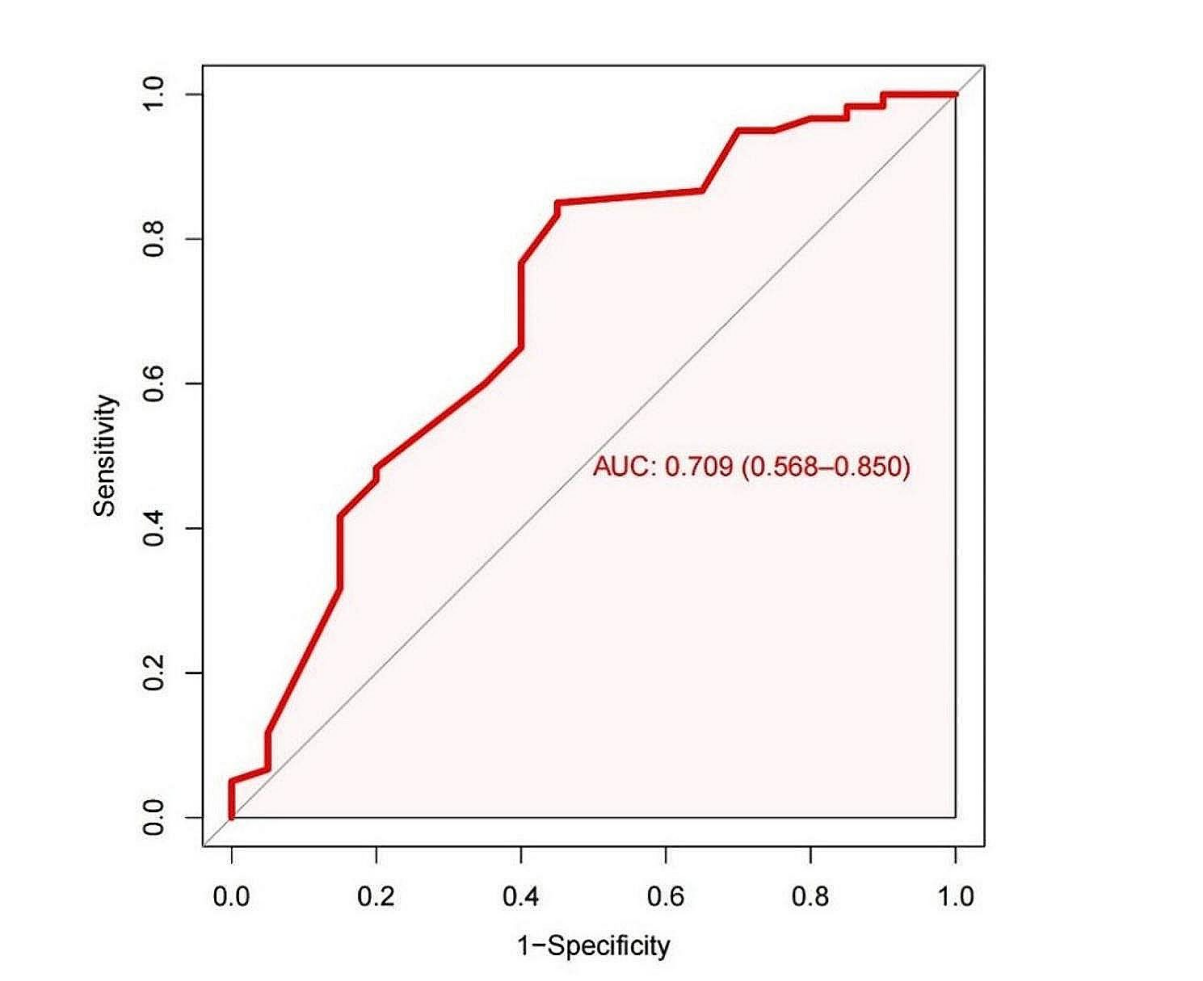
A receiver operating characteristic analysis revealed a moderate accuracy of predicting the improvement of LVEF by left ventricular end-diastolic diameter (LVDd) with a cutoff of 59 mm (sensitivity: 85.0%, specificity: 55.0%, area under curve: 0.709)

In the univariate analysis (Table 3), age, LVDd, e-GFR and LVZs were associated with LVEF improvement. From the Youden index analysis, the best cutoff of LVDd was 59 mm (sensitivity: 0.850, specificity: 0.550, area under the curve: 0.709) (Fig. 1). After *P* < 0.10 of univariate analysis was included in multivariate analysis (Table 3), LVDd (OR = 0.85; 95% CI: 0.76–0.95, *P* = 0.005) and LVZs (OR = 0.26; 95% CI: 0.07–0.96, *P* = 0.043) were still significantly associated with improvement of LVEF. LVDd and LVZs were independent predictors of improvement in LVEF after catheter ablation in patients with atrial fibrillation complicated with heart failure.

**Table 3:** Association of patient characteristics with improvement of LVEF : univariate and multivariate regression analysis

	Univariate			Multivariate	
Variable	OR(95% CI)	*P* value		OR(95% CI)	*P* value
Age(year)	0.94(0.88–0.99)	**0.024**		0.97(0.90–1.04)	0.324
Female n(%)	0.64(0.23–1.84)	0.410			
Weight(kg)	1.03(0.99–1.07)	0.138			
BMI(kg/m^2^)	1.08(0.92–1.26)	0.349			
AF duration (months)	0.99(0.97–1.01)	0.311			
LVEF(%)	0.92(0.82–1.03)	0.133			
LVDd(mm)	0.86(0.78–0.95)	**0.004**		0.85(0.76–0.95)	**0.005**
LAD(mm)	0.99(0.88–1.10)	0.820			
Hypertension(%)	1.00(0.36–2.81)	1.000			
Diabetes(%)	0.56(0.11–2.78)	0.475			
CHD(%)	0.92(0.29–2.93)	0.883			
Previous stroke n(%)	1.49(0.45–4.96)	0.520			
NT-pro BNP(pg/ml)	1.00(1.00–1.00)	0.221			
Hb(g/L)	1.02(1.00-1.06)	0.105			
eGFR(ml/min/1.73m^2^)	1.04(1.01–1.07)	**0.009**		1.01(0.98–1.06)	0.395
TC(mmol/L)	1.41(0.75–2.66)	0.284			
TG(mmol/L)	0.75(0.24–2.33)	0.617			
LDL-C(mmol/L)	0.97(0.90–1.05)	0.470			
HDL-C(mmol/L)	8.91(0.69–114.90)	0.094		7.35(0.26-208.54)	0.243
ACEI/ARB/ARNI n(%)	0.91(0.28–2.96)	0.879			
$\beta$-block n(%)	0.53(0.14–2.04)	0.354			
Diuretic n(%)	0.68(0.23–1.99)	0.477			
SGLT-2 n(%)	0.56(0.11–2.78)	0.475			
Digoxin n(%)	0.71(0.18–2.81)	0.621			
EAT volume(cm^3^)	1.00(0.99–1.02)	0.816			
LA-EAT volume (cm^3^)	0.99(0.96–1.03)	0.546			
LVZs n(%)	5.04(1.68–15.13)	**0.004**		0.26(0.07–0.96)	**0.043**
Recurrence of AF n(%)	1.11(0.31–3.99)	0.869			

I group = improve group, NI group = nonimprove group, PeAF = persistent atrial fibrillation, IHD = ischemic heart disease, ACEI = angiotensin-converting enzyme inhibitor, ARB = angiotensin receptor blocker, ARNI = angiotensin receptor neprilysin inhibition, SGLT-2i = sodium-dependent glucose transporters 2 inhibitor, AADs = antiarrhythmic drugs, EAT = epicardial adipose tissue, LVEF = left ventricular ejection fractions, LAD = left atrial diameter, LVDd = left ventricular end diastolic diameter, LVZs = low voltage zones

**Fig. 2:**
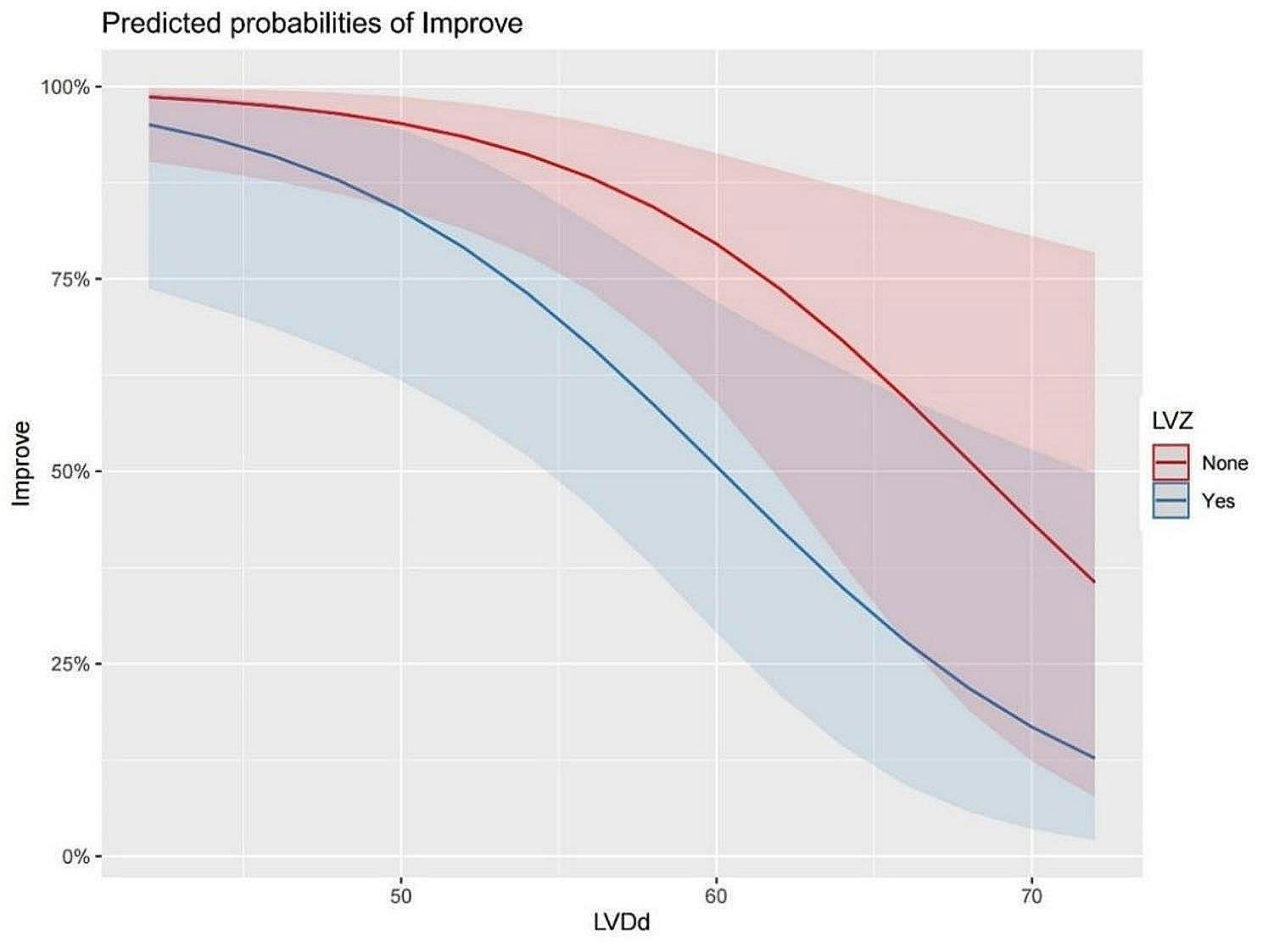
Prediction of the probabilities of LVEF improvement

Figure 2 shows that, on the one hand, the possibility of postoperative LVEF improvement with increasing LVDd gradually decreased. On the other hand, patients with no low voltage zones in the left atrium had a higher probability of improvement than those with low voltage zones.

We measured the size of the low voltage zone and its proportion in the left atrium in 33 patients in this study, there was no difference in the size of the low voltage area between the two groups [19.0 (9.0,22.5) cm^2^ vs. 21.0 (8.0,30.0) cm^2^, *P* = 0.913] and the same is true for the results of the proportion of low voltage zone [10.0(4.3,14.2) % vs. 11.1(4.6,16.0) %, *P* = 0.662]. A receiver operating characteristic analysis determined that the suitable cutoff value for LVZs area was 14.5 cm^2^ (sensitivity: 64.3%, specificity: 47.4%, area under curve: 0.511) and the suitable cutoff value for its proportion in the left atrium was 14.5% (sensitivity: 42.9%, specificity: 73.7%, area under curve: 0.545).(Table S1).

### Value of radiofrequency ablation for atrial fibrillation

**Fig. 3:**
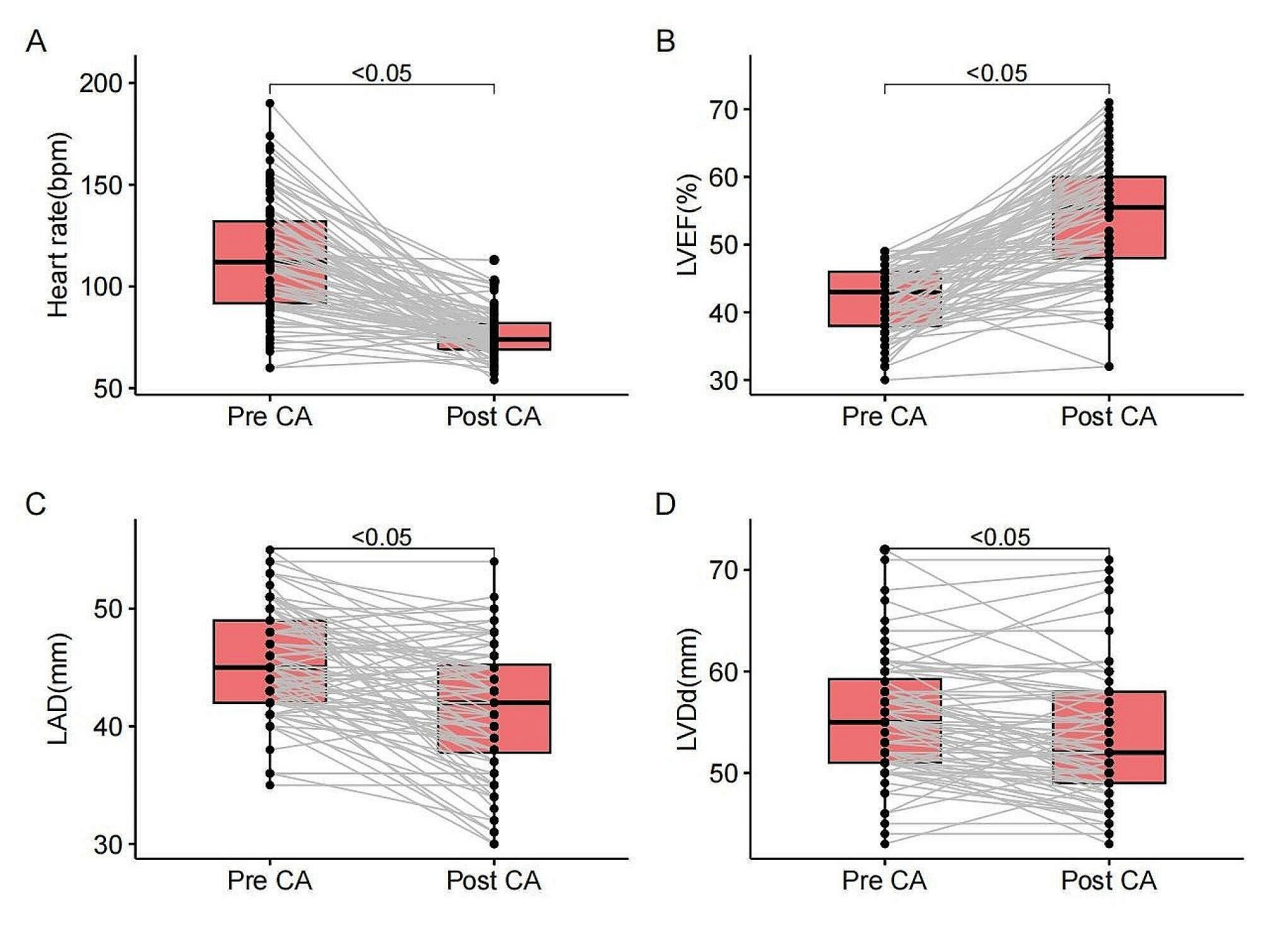
Structural and functional changes in the heart (LVEF = left ventricular ejection fraction, LAD = left atrial diameter, LVDd = left ventricular end diastolic diameter, CA = catheter ablation)

Figure 3 shows the changes in the structure and function of the patient’s heart after radiofrequency ablation. LVEF (42 ± 5% vs. 54 ± 9%, *P* < 0.05) improved, and LVDd (55 ± 6 mm vs. 53 ± 6 mm, *p* < 0.05), LAD (45 ± 5 mm vs. 41 ± 6 mm, *p* < 0.05), and HR (114 ± 28 bpm vs. 76 ± 12 bpm, *p* < 0.05) were all significantly lower.

## Discussion

This study clarified the predictors of improved cardiac function after ablation in AF patients with low ejection fraction, which provided the early identification of AF-TCM. The main findings are as follows: (1) LVDd < 59 mm and no low voltage zones in the left atrium are independent predictors of left ventricular ejection fraction improvement after AF ablation. (2) Radiofrequency ablation of atrial fibrillation has significant benefits in improving left ventricular systolic function in patients, restoring the sinus rate and reducing the ventricular rate in patients. (3) The volume of epicardial adipose tissue has no apparent relationship with the improvement of LVEF in this study.

Numerous studies, including ours, have found that LVDd is an independent predictor of LVEF improvement. Ukita et al. found that LVDd < 53 mm might be an independent predictor of LVEF improvement after catheter ablation of persistent AF in HFrEF patients[18]. Another study [9, 19, 20] demonstrated that freedom of AF recurrence and absence of ventricular late gadolinium enhancement (LGE) on cardiac magnetic resonance imaging (MRI) predict improvement of LV dysfunction after AF ablation. In this study, we found that 75.0% of patients improved LVEF after ablation, and the LVDd of these patients was smaller than that of the nonimproved group. Left ventricular enlargement was more evident in the nonimproved group. The most widely accepted explanation is that left ventricular enlargement is a compensatory mechanism for LV systolic dysfunction [21]. This compensatory mechanism can maintain the normal ejection fraction in a short time, but with the further expansion of the left ventricle, this compensation gradually becomes an irreversible injury. Through further analysis, we considered LVDd < 59 mm as an independent predictor of improvement in LVEF. After effective control of AF, these patients can recover better cardiac function in the short term. We only considered early ejection fraction improvement in this study. Despite promising early outcomes, rapid deterioration of systolic function with recurrence of tachycardia and late sudden death have been reported, raising the possibility that the left ventricular myocardial substrate remains diseased despite normalization of overall systolic function [22, 23].

Conversely, patients in the group without improvement in cardiac function had more left atrial low voltage zones; this may be related to heart failure causing electrical and structural changes in the left atrium and the whole heart. Other possible causes include chronic atrial distraction and various neurohumoral hormone activation. Atrial stretch alters cellular gene expression and activates the channel, in part through stretching, leading to the initiation of cell hypertrophy, changes in ionic transmembrane current and action potential duration, and the opening of angiotensin II synthesis. The activation of neurohormones in renin-angiotensin-aldosterone systems is a potent stimulator of myocardial fibrosis. AF and HF together accelerate this process, and the low voltage zone precisely reflects the degree of atrial fibrosis. The nonimproved group had more severe heart failure, with deeper atrial fibrosis and more low-voltage areas. In a previous study, Kirstein found that the extent of LA fibrosis and LVEF response are inversely correlated, with no treatment effect beyond 35–40% LA fibrosis [24]. We further measured the size of the low voltage zone and its proportion in the total left atrial area, but neither was a good predictor of improvement in left ventricular function after atrial fibrillation ablation. This may have something to do with our smaller number of cases.

With our understanding of EAT rapidly increasing in recent years [25], basic research [26, 27] has shown that EAT can lead to fibrosis through fatty infiltration, profibrosis, and inflammatory responses (autocrine and paracrine secretion of proinflammatory cytokines). A previous study confirmed that prominent amounts of EAT could induce atrial fibrosis in rats [28]. Moreover, the distribution and volume of LA-EAT are closely related to left atrial fibrosis. Therefore, we measured EAT and LA-EAT volume, but we have yet to find a relationship between EAT and LVEF improvement in this study. Undeniably, EAT and LVZs both reflect the degree of atrial fibrosis, and their spatial distribution is coincident [13]; this provides us with a method for the noninvasive measurement of LVZs.

At present, radiofrequency ablation of atrial fibrillation is widely used in patients with heart failure, and several studies have proven that it has obvious advantages in improving the quality of life of patients with atrial fibrillation and reducing the incidence of cardiovascular events [9, 29–31], which is consistent with our research.

### Limitations

The limitations of this study are as follows: (1) This work is a single-center study with an insufficient study population. (2) This study had only a 1-year follow-up, which could not evaluate the effect of recurrence of late AF on LVEF improvement. The 24-h Holter monitoring reflected heart rate changes and AF recurrence more accurately than a single 12-lead ECG. Nevertheless, we did not have Holter data for all patients.

## Conclusions

Our study provides additional evidence that LVDd < 59 mm and no low voltage zones in the left atrium can be used to jointly predict the improvement of left ventricular ejection fraction after atrial fibrillation ablation, allowing early identification of those patients with AF-TCM who require closer clinical monitoring and intensive HF therapy.

### Electronic supplementary material

Below is the link to the electronic supplementary material.

Supplementary Material 1

## Data Availability

Data for this study can be applied to the corresponding author upon reasonable request.
