# Supplementary material for: Predictors of improvement in left ventricular systolic function after catheter ablation in patients with persistent atrial fibrillation complicated with heart failure
Source: BMC Cardiovasc Disord. 2024 Mar 23;24:178. doi: 10.1186/s12872-024-03840-z (PMC10960375; doi:10.1186/s12872-024-03840-z)
Supplement: Supplementary file 1 — Supplementary Material 1 [file 12872_2024_3840_MOESM1_ESM.docx]

| Table S1 The size of the low voltage zones and its proportion in the left atrium in 33 patients | | | |
| --- | --- | --- | --- |
|  | I group(n = 19) | NI group(n = 14) | *P* -value |
| LVZs area (cm^2^) | 19.0(9.0,22.5) | 21.0(8.0,30.0) | 0.913 |
| LVZs area / LA area (%) | 10.0(4.3,14.2) | 11.1(4.6,16.0) | 0.662 |
| I group = improve group, NI group = nonimprove group, LVZs = low voltage zones, LA = left atrium. | | | |


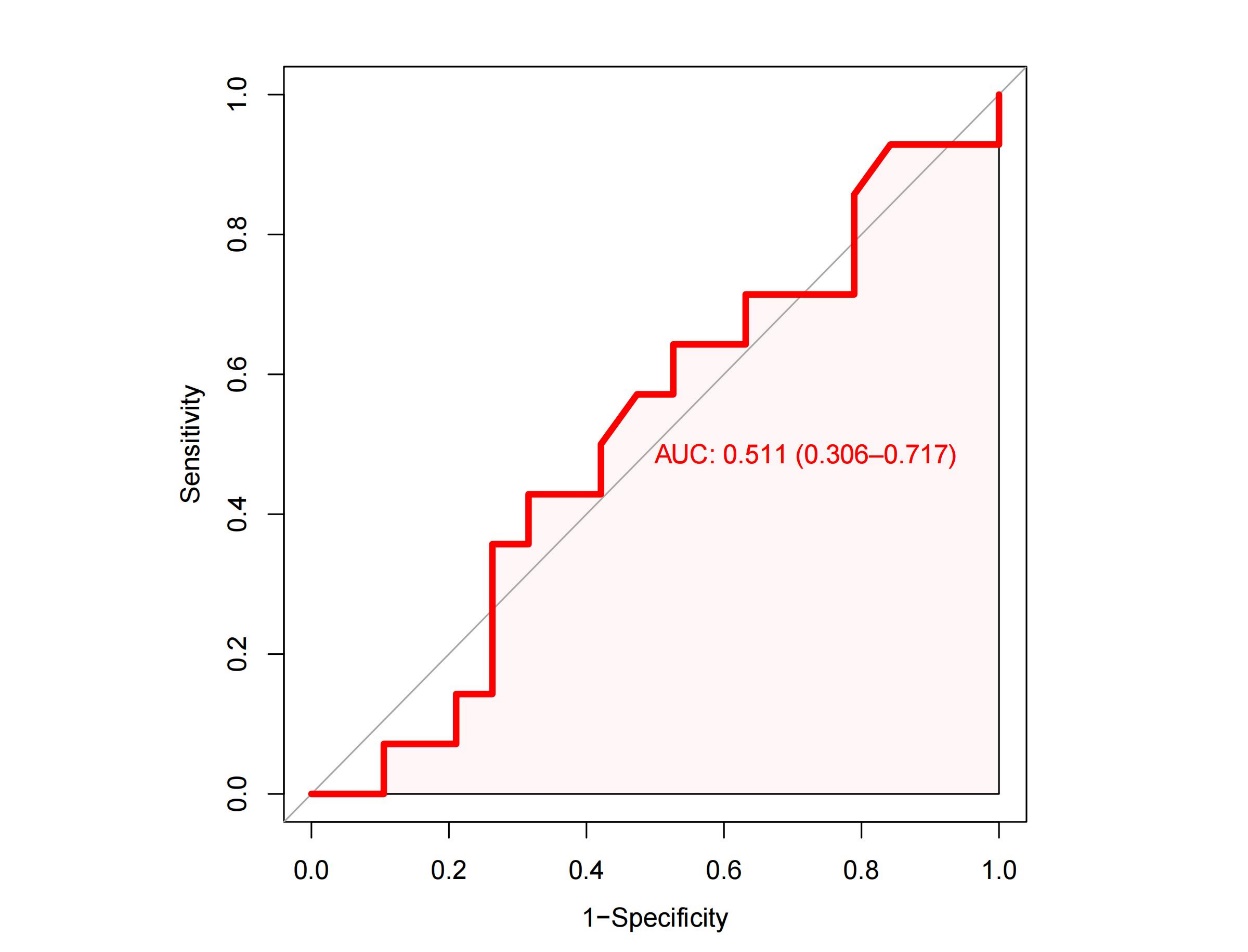


Figure S1 Analysis of receiver operating characteristics showed that the area of LVZ was low accurate in predicting improvement in LVEF, with a cut-off value of 14.5 cm^2^ (sensitivity: 64.3%, specificity: 47.4%, area under the curve: 0.511).


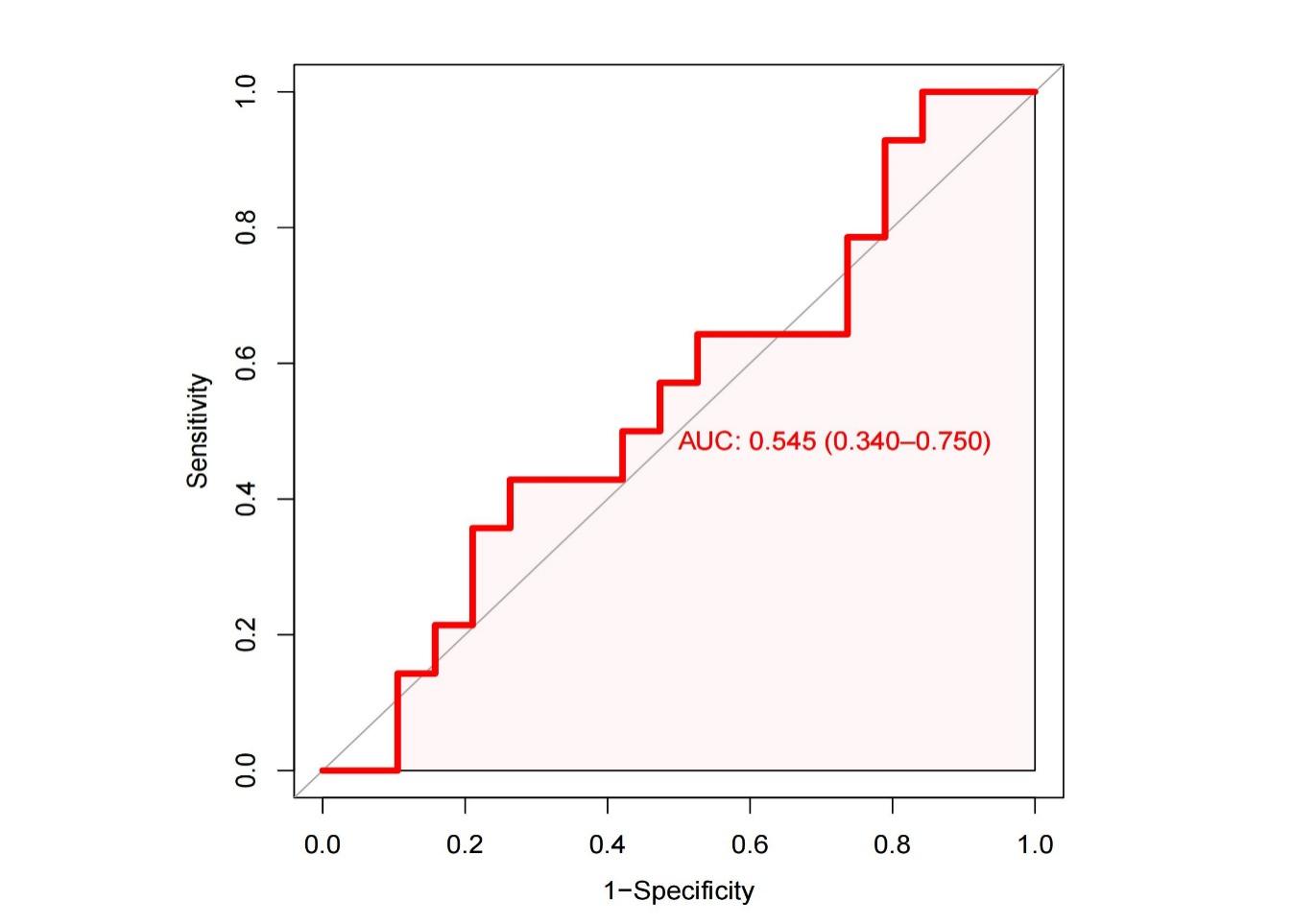


Figure S2 Analysis of receiver operating characteristics showed that the proportion of low voltage area in the left atrium was low accurate in predicting improvement in LVEF, with a cut-off value of 14% (sensitivity: 42.9%, specificity: 73.7%, area under the curve: 0.545).
